# What Defines Quality of Life for Older Patients Diagnosed with Cancer? A Qualitative Study

**DOI:** 10.3390/cancers14051123

**Published:** 2022-02-22

**Authors:** Petronella A. L. (Nelleke) Seghers, Jolina A. Kregting, Lieke H. van Huis-Tanja, Pierre Soubeyran, Shane O’Hanlon, Siri Rostoft, Marije E. Hamaker, Johanneke E. A. Portielje

**Affiliations:** 1 Department of Geriatric Medicine, Diakonessenhuis, 3582 KE Utrecht, The Netherlands; 2 Department of Internal Medicine, Canisius Wilhelmina Ziekenhuis, 6532 SZ Nijmegen, The Netherlands; j.kregting@cwz.nl; 3 Department of Internal Medicine, Diakonessenhuis, 3582 KE Utrecht, The Netherlands; lvhuis@diakhuis.nl; 4 Department of Medical Oncology, Institut Bergonié, Université de Bordeaux, 33076 Bordeaux, France; p.soubeyran@bordeaux.unicancer.fr; 5 Department of Geriatric Medicine, St Vincent’s University Hospital, D04 T6F4 Dublin, Ireland; shaneohanlon@svhg.ie; 6 Department of Geriatric Medicine, University College Dublin, D04 V1W8 Dublin, Ireland; 7 Department of Geriatric Medicine, Oslo University Hospital, 0424 Oslo, Norway; siri.rostoft@medisin.uio.no; 8 Institute of Clinical Medicine, University of Oslo, 0318 Oslo, Norway; 9 Department of Medical Oncology, Leiden University Medical Center-LUMC, 2333 ZA Leiden, The Netherlands; j.e.a.portielje@lumc.nl

**Keywords:** cancer, older patients, geriatric oncology, quality of life

## Abstract

**Simple Summary:**

Quality of life has a different meaning for every individual. In older patients with cancer, quality of life is important because anti-cancer treatment may influence their quality of life. In order to assess the aspects of quality of life that matter most to older patients with cancer, we interviewed 63 patients. We used both open-ended questions and asked them to select the most important items from a predefined list: cognition, contact with family or with community, independence, staying in your own home, helping others, having enough energy, emotional well-being, life satisfaction, religion and leisure activities. Physical functioning, social functioning, physical health and cognition are important components of quality of life. In conclusion, maintaining cognition and independence, staying in one’s own home, and maintaining contact with family and community appear to be the most important aspects of quality of life for older patients with cancer. These aspects should be included when making a shared treatment decision.

**Abstract:**

The treatment of cancer can have a significant impact on quality of life in older patients and this needs to be taken into account in decision making. However, quality of life can consist of many different components with varying importance between individuals. We set out to assess how older patients with cancer define quality of life and the components that are most significant to them. This was a single-centre, qualitative interview study. Patients aged 70 years or older with cancer were asked to answer open-ended questions: What makes life worthwhile? What does quality of life mean to you? What could affect your quality of life? Subsequently, they were asked to choose the five most important determinants of quality of life from a predefined list: cognition, contact with family or with community, independence, staying in your own home, helping others, having enough energy, emotional well-being, life satisfaction, religion and leisure activities. Afterwards, answers to the open-ended questions were independently categorized by two authors. The proportion of patients mentioning each category in the open-ended questions were compared to the predefined questions. Overall, 63 patients (median age 76 years) were included. When asked, “What makes life worthwhile?”, patients identified social functioning (86%) most frequently. Moreover, to define quality of life, patients most frequently mentioned categories in the domains of physical functioning (70%) and physical health (48%). Maintaining cognition was mentioned in 17% of the open-ended questions and it was the most commonly chosen option from the list of determinants (72% of respondents). In conclusion, physical functioning, social functioning, physical health and cognition are important components in quality of life. When discussing treatment options, the impact of treatment on these aspects should be taken into consideration.

## 1. Introduction

When a patient is diagnosed with cancer, treatment decisions ideally include evaluation of outcomes such as survival, the side-effects of treatment and patient-related outcomes such as quality of life. For shared decision making, knowing what matters most in life, rather than simply asking whether a patient would want a certain treatment, will give more clarity regarding treatments that should be considered or avoided for an individual patient [1]. To optimally counsel patients, it is important to know their priorities and how they value outcome options in relation to each other [2]. In a clinical setting, this also means understanding what quality of life means to the individual.

The World Health Organisation defines quality of life as “an individual’s perception of their position in life in the context of the culture and value systems in which they live and in relation to their goals, expectations, standards and concerns. It is a broad ranging concept affected in a complex way by the person’s physical health, psychological state, personal beliefs, social relationships and their relationship to salient features of the environment” [3]. This definition implies that quality of life has a different meaning for each individual, in the context of personal goals and expectations.

Most instruments that measure quality of life are generated for research purposes to assess the effect of treatment or disease on quality of life [4,5]. These questionnaires try to capture quality of life in measurable variables, such as functional ability and presence or absence of specific symptoms. In a clinical setting, these questionnaires may be of limited help since they measure the presence of deficits that potentially impair quality of life, but they lack the ability to identify which items really matter to the individual patient in maintaining quality of life [6].

What really matters to a patient may shift in older patients with cancer compared to younger patients; for example, functioning or other aspects of quality of life may be more important to them than survival. Moreover, as the adverse events may be of greater significance [7] and clinical benefit could be more limited than in younger patients, older patients have a different balance between benefits and risks [8,9]. This may also influence their priorities [8,9]. In 2020, 52% of new cancers were diagnosed in patients older than 70 years [10]. As the prevalence of older adults with cancer is increasing [10], understanding what matters most to older patients with cancer becomes increasingly important.

Therefore, we asked patients aged 70 and older with cancer what constituted quality of life to them and which factors could change the quality of life they experienced.

## 2. Materials and Methods

### 2.1. Population and Sample

This study is a prospective single-centre study conducted at the Diakonessenhuis Utrecht, a community hospital. Patients diagnosed with cancer or treated for cancer during the last two years and aged >70 years at the time of inclusion, were asked to participate. They were enrolled during a consultation with their treating oncologist or when they were receiving treatment in the outpatient clinic. Patients were only approached if they came for a routine visit or check-up, if their situation was stable (as judged by their health care provider) and if they did not have cognitive impairment, depressed mood or anxiety issues. These exclusion criteria were chosen because participation was considered too burdensome for this group of patients. The study was reviewed by the ethics committee and it received a waiver for full ethics review, consecutively the protocol was approved by the local research committee.

### 2.2. Patient Interview

After explaining the study concept to patients, they provided written informed consent. The subsequent interview was done by telephone at a time that suited the patient. All interviews were conducted by one interviewer (NS). All interviews lasted between 15 and 45 min, and patients were given as much time as needed to respond. A written transcript was made during the interview and conversations were not recorded.

During interviews, patients were asked to respond to open ended-questions: What makes life worthwhile? What does quality of life mean to you or what defines it? What could affect your quality of life? If they answered with broad or vague terms, they were asked to specify as much as possible.

Next, patients were asked to select the top five important determinants of quality of life out of a list of thirteen questions (Appendix A). This list was based on the items of the Functional Assessment of Cancer Therapy-General (FACT-G) and the European Organization for Research and Treatment for Cancer Core Quality of Life Questionnaire-30 (EORTC-QLQ-C30) [4,5]. Two symptoms were selected (fatigue and pain) in addition to relevant items from the general health and functional subscales. Patients were encouraged to discuss each item and what it meant to them, and then to select their top five. If they were unable to limit their choices to five, additional answers were allowed.

Afterwards, patients were asked about their current ability to perform the basic and instrumental activities of daily living (ADL [11] and IADL [12], respectively), and their education (high, medium or low), partner status and usage of a walking aid.

In addition to the patient interview, the following data were extracted from the patient’s electronic health file: age, comorbidity, tumour type, stage of disease and received treatment. Based on the information extracted, the Charlson comorbidity index [13] was completed for each patient.

### 2.3. Data Synthesis and Analysis

From the open-ended questions, answers were independently categorized by the two authors (NS and JK). An initial classification was done based on the categories in Appendix A that covered five main domains: physical health, physical functioning, psychological and cognitive functioning, social functioning and meaningful life. When necessary, the help of a third author (MH) was requested. Categories were refined and added as needed until consensus on the classification was reached for 14 categories. These 5 domains and 14 categories can be found in Table 1.

The results were reported using descriptive data. For normally distributed data, means with standard deviations were used; and, for non-normal distributions medians with range were used. To make comparisons between respondents’ answers and between male and female patients, a chi-square test (category chosen (yes-no)) [14] was used. Statistically significant *p*-values were smaller than 0.05.

## 3. Results

### 3.1. Patient Characteristics

Between September 2020 and April 2021, 63 patients were interviewed (Table 2). The median age was 76 (range 70–92 years) and 48% of the participants were male. Forty-three patients (68%) had a partner. The median Charlson comorbidity index was 1 (range 0–6): 24% of patients were impaired in ADL and 33% were impaired in iADL. A walking aid was used by 30% of the participants.

The most common form of cancer was breast cancer (*n* = 16, 25%), followed by multiple myeloma (*n* = 10, 16%). Two-thirds of patients (*n* = 42) were undergoing or had completed chemotherapy and 26 patients had undergone surgery.

### 3.2. Open-Ended Questions

The interview started with the question: What makes life worthwhile? In their answers, 86% of the participants mentioned items that were categorized in the domain of social functioning, with 84% mentioning items in the domain of physical functioning. The categorization of answers into multiple domains was possible.

**Figure 1:**
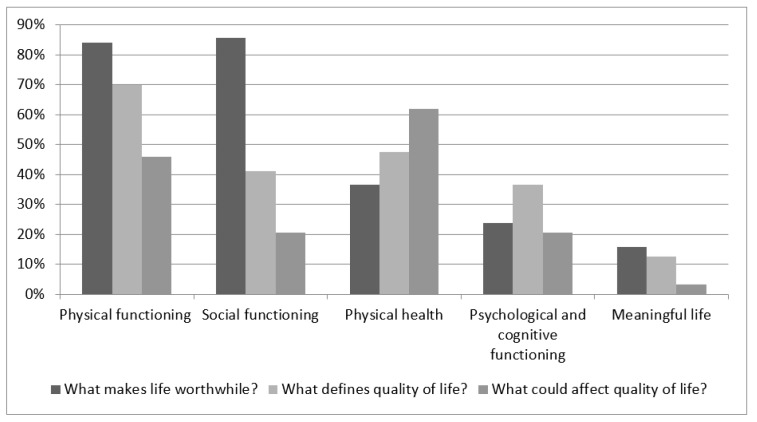
Open ended questions. Proportion of participants mentioning each domain of a question: What makes life worthwhile? What defines quality of life for you? What could affect (improve or decrease) your quality of life?

Next, patients were asked to define quality of life. The most common answers were categorized in the physical functioning domain (70%) and the physical health domain (48%). These items were also the most important factors that could affect current quality of life and they were mentioned by 46% and 62% of the participants, respectively. Social functioning was mentioned in 41% of the answers to the question to define their quality of life (Figure 1). For more details see Appendix B.

### 3.3. Selecting Top FIVE Priorities

From the predefined list, patients selected cognition (72%), contact with family (70%), independence (57%) and staying in your own home (48%) as the most important factors for quality of life (Table 3).

There were no statistically significant differences between male and female participants. Both sexes gave the highest priority to cognition, contact with family and independence (Table 4). There was a non-significant trend for females to prioritize staying in one’s own home (55% vs. 37% of males, *p* = 0.16) and for males to prioritize contact with the community (47% vs. 30% of females, *p* = 0.18).

Presenting the predefined list yielded other priorities than the open-ended questions (Table 3). Cognition was most frequently selected from the list (72%), but it was mentioned by only 17% of patients in the open-ended questions (*p* < 0.05). A similar difference was seen for staying in your own home (48% versus 3%, *p* < 0.05) and having enough energy (28% versus 10%, *p* < 0.05). Independence was equally reported by both sexes (57% and 48%, *p*-value 0.32). Leisure activities were rarely selected in the predefined list (13%) and often mentioned in the open-ended questions (49%, *p* < 0.05) in which many people mentioned a specific hobby. Overall, patients mentioned a median of two to four categories in the open-ended questions.

## 4. Discussion

In this qualitative interview study, we interviewed 63 patients aged above 70 years who were undergoing or who had received treatment for cancer during the past two years. In the open-ended questions, aspects of physical functioning, physical health and social functioning were mentioned most frequently as components that defined quality of life and made life worthwhile. From a predefined list, cognition, contact with family, independence, staying in your own home, and contact with the community were selected most frequently to define quality of life.

Although quality of life and what makes life worthwhile are closely related questions, they are not the same and they yielded different answers. Social functioning was more often mentioned in what makes life worthwhile (84% vs. 41%). This makes sense if we imagine a patient who is facing declining health and a decline in quality of life but still considers their life worthwhile due to the family that surrounds them. It is, however, important to realize that the exact phrasing of the question may influence the answer, particularly because patients may have their own understanding of common terms such as quality of life, which may require additional explanation. The prioritized aspects of quality of life in open-ended questions and choosing from predefined answer options yielded different results. This may be because patients did not: think of certain options, realize that cancer treatment may affect aspects such as cognition, or realize that their highly valued leisure activities may depend on underlying factors, such as preservation of cognitive and physical function. However, if presented with the predefined answers with aspects such as cognition and having enough energy, patients prioritized these over being able to perform leisure activities. Interestingly, independence was equally chosen in the open and the closed questions. This could be because patients do realize that autonomy is important for their quality of life, even if it is not yet mentioned as an option.

Another explanation for the difference between the types of questions is that the predefined answers may fail to reflect the entire definition and structure of quality of life, including the connections between the values for an individual. The relation between the various components may be explained by a concept described earlier in another context by Maslow [15]. He described five levels of needs in a pyramid shape, ranging from the very basic needs of food, warmth and shelter, to the highest level of self-fulfilment [15]. Each level of the pyramid requires the level below to be fulfilled. In quality of life, basic needs that may be compulsory for an individual are aspects such as cognition, good health and enough energy. Further up the pyramid are more complex activities and values such as helping others or performing leisure activities. This is why questionnaires that list possible items of quality of life, without giving the option to explain or rate the various items in relation to each other, may not be enough to fully understand the patient’s quality of life. It is important to know exactly what patients value in life because this will help to select the best suited treatment. For example, a patient could highly value caring for a sick partner and therefore state that they prefer to live as long as possible, including being willing to accept all treatment related toxic effects. If, however, confronted with a loss of physical condition that is considered necessary to care for their sick partner, they may choose a different treatment. Hence, it may be helpful to ask patients open-ended questions to define quality of life and ask them to specify which underlying components of their answer are most important and why. Further research is needed in this area.

Although the comparison with prior research is complicated by the fact that each study uses slightly different types and numbers of domains, our finding that living independently and maintaining one’s psychological, physical and social function are important components of quality of life, appear to be in line with earlier study results [16,17,18,19]. Previous research in older patients has shown that few patients are willing to prioritize survival over cognition or avoidance of severe functional impairment [20,21]. Even though a myriad of publications over the past twenty years has advocated the importance of obtaining information on the impact of treatments on specific quality of life determinants such as functioning and cognition [20], this type of information is still lacking. This may be due in part to the way quality of life is studied in clinical trials.

Looking at our study, all answers that patients gave can be found in commonly used quality of life questionnaires such as the EORTC-QLQ-C30 or the FACT-G, which are developed to measure the impact of a treatment on quality of life in the study arm and to compare it to quality of life in the control arm in a clinical trial [4,5]. These questionnaires thus seem to suffice in terms of comprehensiveness. However, they lack the possibility for weighting the relative importance of each item and of individual definitions. An individualised measure such as the SEIQOL-DW (Schedule for the Evaluation of Individual Quality of Life- Direct Weighting [22,23] asks patients to nominate their quality of life domains freely and to rate and weight these thereafter. These individualised measurements are, however, incapable of measuring the treatment effect on quality of life beyond the individual level due to the heterogeneous answers. A solution for this gap could be for trials to not only report on the overall quality of life score and provide group summaries but to also report in more detail on subscales and inter-individual variation in the quality of life trajectory over time [24]. This information could be translated to the impact on the patient’s individual quality of life by the clinician. Further research is needed on the reporting of quality of life in clinical trials in more detail and how to incorporate these results into clinical practice.

This study has some limitations. First of all, patient priorities and values may change over time and depend on the context of the patient. Patients included in this study were independent, overall highly educated and had either recently completed their cancer treatment or were in the midst of it. We do not know if our results are generalizable to patients who are newly diagnosed, less educated, long-term survivors or more dependent. Another contextual factor that may have influenced the values that patients expressed is the fact that the Dutch government held several lock-downs due to COVID-19, resulting in limitations in possibilities to carry out usual activities. Secondly, the categories were made by two authors based on consensus and other ways of categorization would have been possible. However, as our results are in line with previous research [16,20], we do not think the results would have been significantly different if categories had been chosen differently. Finally, we only categorized explicitly mentioned items to minimize interpretation bias. This may have led to underreporting of aspects that patients do consider important but did not explicitly mention. In clinical practice, it is therefore important to also ask patients for reasons why certain aspects are considered important.

## 5. Conclusions

In conclusion, maintaining cognition and independence, staying in one’s own home, and contact with both family and community appear to be the most important aspects of quality of life for older patients with cancer. These aspects should be measured and reported in detail in clinical trials to acquire the information that patients and doctors need to make a shared treatment decision. The way of asking a patient about their quality of life influences the answer, therefore, this should be taken into consideration when assessing a patient’s individual definition of quality of life.

## Figures and Tables

**Table 1:** Domains, categories and definitions. The following categories are made to classify the open-ended answers.

Domain	Name of the Category	Definition of the Category
Physical health	Energy	Have enough energy and good physical condition
	Health	No physical complaints, no problems of the disease or treatment bothering you, maintaining good health, control of the disease, have no other disease, etc.
Psychological and cognitive functioning	Cognition	Have no trouble thinking, remembering or concentrating
	Well-being	Emotional well-being, not being anxious, not being depressed, no excessive worrying, calmness of mind
	Not worry about others	Prosperity of your friends and family, so you don’t have to worry about them
Physical functioning	Independence	Being independent, freedom to go where you want, no need for help from other people, good mobility, etc.
	Leisure activities	Being able to perform the activities that you want, that give you joy and satisfaction, e.g., hobbies, holiday, nature, food.
	Staying in your own home	Being able to live in your own home and not move to a nursing home
Social functioning	Partner	Being together with a partner or having the comforting memories of a good marriage or relationship
	Family (including partner)	The support or presence of other family
	Community	Support from friends, acquaintances, and other people from the community
	Helping others	Being able to help others
Meaningful life	Religion	Support of your religion
	Good life	The satisfaction of your life being important, looking back on a good life
Other	Finances	Having enough money without having to worry too much

**Table 2:** Demographics of the population.

Characteristics	*n* (%)
Sex	
Male	30 (48%)
Female	33 (52%)
Age median (range)	76 years (70–92)
Partner status	
Widow(er)	16 (25%)
Current partner	43 (68%)
Impaired in basic activities of daily living	15 (24%)
Impaired in instrumental activities of daily living	21 (33%)
No walking aid	43 (68%)
Charlson Comorbidity Index (in addition to cancer)	1 (0–6)
Education	
University or higher education	29 (46%)
Vocational training	11 (17%)
Primary/secondary education or less	8 (13%)
Missing	15 (24%)
Types of cancer	
Breast cancer	16 (25%)
Multiple myeloma	10 (16%)
Prostate cancer	9 (14%)
Chronic Lymphocytic	4 (6%)
Chronic lymphocytic leukaemia	
Lymphoma (various types)	7 (11%)
Ovarian cancer	3 (5%)
Colorectal cancer	9 (14%)
Other gastrointestinal tumours	5 (8%)
Disease stage	
Stage I	9 (14%)
Stage II	4 (6%)
Stage III	9 (14%)
Stage IV	27 (43%)
Other (hematologic)	14 (22%)
Received treatment (multiple options per patient possible)	
Chemotherapy	42 (67%)
Surgery	26 (41%)
Targeted therapy	21 (33%)
Immunotherapy	18 (29%)
Hormone therapy	18 (29%)
Radiation therapy	17 (27%)
Corticosteroids	17 (27%)

**Table 3:** Selection of categories in predefined answer options vs. open-ended question regarding quality of life.

Categories	Open-Ended Questions (*n* = 63)	Predefined Options (*n* = 60)	*p*-Value
Cognition	11 (17%)	43 (72%)	<0.001
Family (including partner)	16 (27%)	42 (70%)	<0.001
Independence	30 (48%)	34 (57%)	0.32
Staying in your own home	2 (3%)	29 (48%)	<0.001
Community	15 (24%)	24 (40%)	0.05
Helping others	7 (11%)	21 (35%)	0.002
Energy	6 (10%)	17 (28%)	0.01
Health	27 (43%)	16 (27%)	0.06
Good life	4 (6%)	15 (25%)	0.004
Well-being	13 (21%)	13 (22%)	0.89
Religion	4 (6%)	10 (17%)	0.07
Leisure activities	31 (49%)	8 (13%)	<0.001
Financial worries	3 (5%)	**	x

** not asked in the top five selection.

**Table 4:** Gender differences in category selection.

Categories	Male (*n* = 30)	Female (*n* = 33)	*p*-Value
Cognition	23 (77%)	20 (61%)	0.17
Family (including partner)	21 (70%)	21 (64%)	0.59
Independence	15 (48%)	20 (59%)	0.40
Staying in your own home	11 (37%)	18 (55%)	0.16
Community	14 (47%)	10 (30%)	0.18
Helping others	9 (30%)	12 (36%)	0.59
Energy	7 (23%)	10 (30%)	0.53
Health	7 (23%)	9 (27%)	0.72
Good life	6 (20%)	9 (27%)	0.50
Religion	6 (20%)	4 (12%)	0.39
Well-being	4 (13%)	9 (27%)	0.17
Leisure	3 (10%)	5 (15%)	0.54

## Data Availability

The data presented in this study are available on request from the corresponding author.

## Appendix A

The following categories are made to classify the open-ended answers, based on the original items that patients selected to be the five highest priorities to determine a good quality of life.

**Table A1:** List of Categories to Classify the Open-Ended Answers.

Name of the Category	Order in Which They Are Asked in the Predefined List
Independence of ADLs **	1
Cognition	2
Religion	3
Well-being	4
Staying in your own home	5
Independence of iADLs **	6
Contact with family	7
Contact with the community	8
Leisure activities	9
Good life (Life satisfaction)	10
Helping others	11
Having enough energy	12
Health	13
No worry about others *	
Finances *	

* New category, added after analysing the open-ended answers. ** Taken together because they were hard to distinguish in the open ended questions.

## Appendix B

Number of participants that mentioned the different categories in the open ended questions. What makes life worthwhile? (worthwhile) What does quality of life mean to you? (definition QoL) What determinants were most important to a good quality of life? (top 5 priority)

**Table A2:** Answers Allocated to Categories.

Categories	Open Question Worthwhile (% of Participants, N = 63)	Open Question Definition QoL (% of Participants, N = 63)	Top 5 Priority (% of Participants, N = 60)
	N (%)	N (%)	N (%)
Family	53 (84%)	44 (70%)	42 (67%)
Leisure	47 (75%)	31 (49%)	8 (11%)
Independence ±	19 (30%)	30 (48%)	34 (54%)
Health *	18 (29%)	27 (43%)	16 (25%)
Community	30 (48%)	15 (24%)	24 (38%)
Well-being	9 (14%)	13 (21%)	13 (19%)
Cognition	3 (5%)	11 (17%)	43 (68%)
Partner **	28 (44%)	7 (11%)	
Helping	9 (14%)	7 (11%)	21 (32%)
Having enough energy	11 (17%)	6 (10%)	17 (25%)
Religion	4 (6%)	4 (6%)	10 (16%)
Good life	7 (11%)	4 (6%)	15 (22%)
Finances **	4 (6%)	3 (5%)	
Home	1 (2%)	2 (3%)	29 (43%)
Worries about others **	7 (11%)	0	

* in the top-5 priority question ‘no pain’ was asked ± in the top 5-priority patients could chose both iADL and ADL dependence ** the category was added during the analysis based on the open-ended questions. In bold are the categories that were answered by at least 40% of the participants.
